# Adipocytes from New Zealand Obese Mice Exhibit Aberrant Proinflammatory Reactivity to the Stress Signal Heat Shock Protein 60

**DOI:** 10.1155/2014/187153

**Published:** 2014-02-05

**Authors:** Tina Märker, Jennifer Kriebel, Ulrike Wohlrab, Volker Burkart, Christiane Habich

**Affiliations:** ^1^Institute for Clinical Diabetology, German Diabetes Center, Leibniz Center for Diabetes Research, the Heinrich-Heine-University Düsseldorf, Auf'm Hennekamp 65, 40225 Düsseldorf, Germany; ^2^German Center for Diabetes Research (DZD e.V.), Düsseldorf, Germany

## Abstract

Adipocytes release immune mediators that contribute to diabetes-associated inflammatory processes. As the stress protein heat shock protein 60 (Hsp60) induces proinflammatory adipocyte activities, we hypothesized that adipocytes of diabetes-predisposed mice exhibit an increased proinflammatory reactivity to Hsp60. Preadipocytes and mature adipocytes from nonobese diabetic (NOD), New Zealand obese (NZO), and C57BL/6J mice were analyzed for Hsp60 binding, Hsp60-activated signaling pathways, and Hsp60-induced release of the chemokine CXCL-1 (KC), interleukin 6 (IL-6), and macrophage chemoattractant protein-1 (MCP-1). Hsp60 showed specific binding to (pre-)adipocytes of NOD, NZO, and C57BL/6J mice. Hsp60 binding involved conserved binding structure(s) and Hsp60 epitopes and was strongest to NZO mouse-derived mature adipocytes. Hsp60 exposure induced KC, IL-6, and MCP-1 release from (pre-)adipocytes of all mouse strains with a pronounced increase of IL-6 release from NZO mouse-derived adipocytes. Compared to NOD and C57BL/6J mouse derived cells, Hsp60-induced formation of IL-6, KC, and MCP-1 from NZO mouse-derived (pre-)adipocytes strongly depended on NF**κ**B-activation. Increased Hsp60 binding and Hsp60-induced IL-6 release by mature adipocytes of NZO mice suggest that enhanced adipocyte reactivity to the stress signal Hsp60 contributes to inflammatory processes underlying diabetes associated with obesity and insulin resistance.

## 1. Introduction

Type 1 and type 2 diabetes account for more than 95% of all diabetes cases worldwide [1]. Although clinical characteristics show clear differences between type 1 and type 2 diabetes, recent developments indicate that the pathogenesis of the two forms of the disease share a number of fundamental features [2]. Comparative analyses particularly revealed chronic, low-grade inflammatory processes such as systemically elevated levels of proinflammatory immune mediators in both types of diabetes [3, 4]. In type 1 diabetes, systemically increased concentrations of inflammatory mediators may promote immune-mediated destruction of autologous pancreatic beta cells [5–7], whereas in type 2 diabetes, increased systemic levels of inflammatory mediators are supposed to contribute to insulin resistance, a major pathogenetic feature of the disease [8–11].

Previous studies identified the adipose tissue as a prominent source of proinflammatory cytokines and chemokines [12, 13]. This finding gains special importance in view of the observations that an increased adipose tissue mass is associated with an elevated risk for type 2 diabetes [14, 15] and an accelerated progression of type 1 diabetes [16, 17]. Further analyses identified the adipocyte population as an important cellular source of proinflammatory mediators within the adipose tissue and implicate that adipocyte-derived mediators promote local inflammatory processes as well as systemic low-grade inflammation [18–20].

Recently, we demonstrated that the (pro-)inflammatory activity of adipocytes is under control of heat shock protein (Hsp) 60 [21–23], a prominent member of the Hsp family [24]. The capacity to specifically bind to adipocytes and to induce the release of proinflammatory cytokines and chemokines qualifies Hsp60 as a candidate for the induction and progression of inflammatory processes associated with the development of diabetes. Initial evidence for a role of Hsp60 in the progression of insulin-deficient/type 1 diabetes came from observations in the nonobese diabetic (NOD) mouse, an animal model of type 1 diabetes which shows aberrant Hsp60 expression in pancreatic beta cells already in the prediabetic phase [25]. In this model, Hsp60-directed intervention was able to attenuate diabetes [26]. Moreover, patients with type 1 diabetes have increased Hsp60 levels [27] and show delayed disease progression after treatment with the Hsp60-derived peptide p277 [28, 29]. Patients with type 2 diabetes also show elevated levels of Hsp60 in their circulation [30]. In obese individuals, Hsp60 plasma concentrations were increased and positively correlated with body mass index and insulin resistance [31].

These observations indicate that Hsp60 stimulates the proinflammatory activity of adipocytes which then might contribute to the (pro-)inflammatory processes associated with diabetes. However, the increased proinflammatory activity observed in (pre-)diabetic individuals may not only be determined by their elevated Hsp60 levels but also by an increased responsiveness of their adipocyte population to the stress protein. Currently, it is not known whether adipocytes from diabetes-prone individuals exhibit an altered Hsp60 responsiveness. We therefore hypothesized that adipocytes of diabetes-predisposed individuals exhibit an increased responsiveness to the (pro-)inflammatory effects of the stress protein Hsp60. To test the hypothesis, we compared the Hsp60 responsiveness of adipocytes from the NOD mouse, as preferred animal model of type 1 diabetes [32, 33], and from the New Zealand obese (NZO) mouse, a model of obesity and the metabolic syndrome [34]. Using adipocytes from C57BL/6J mice as reference mouse strain without diabetes risk, we investigated the decisive events assumed to be involved in Hsp60-mediated adipocyte activation: (1) the binding of Hsp60, (2) the activation of signalling pathways, and (3) the release of inflammatory mediators.

## 2. Materials and Methods

### 2.1. Animals

Normoglycemic mice (blood glucose concentration < 10 mmol/L) of the strains C57BL/6J (female), NOD (female), and NZO (male) at an age of 70 days were obtained from the breeding colonies at the German Diabetes Center. Animal experimentation was performed in accordance with the principles of laboratory animal care and was approved by the local state animal welfare committee.

### 2.2. Adipocyte Isolation and Culture

Preadipocytes were isolated from the visceral adipose tissue depot of the various mouse strains, cultivated, and differentiated to mature adipocytes as previously described [22]. The purity of the cell populations and their differentiation state were confirmed by lipid-specific Oil Red O staining and FACS analyses as described [21].

### 2.3. Antibodies and Reagents

Antibodies directed against *β*-actin and signalling proteins (phospho-ERK1/2 MAPK Thr202/Tyr204, phospho-p38 MAPK, phospho-SAPK/JNK Thr183/Tyr185, phospho-NF*κ*B p65 Ser536) were purchased from Cell Signalling (Danvers, MA, USA). Murine anti-human Hsp60 Abs were from Santa Cruz (Heidelberg, Germany; clones H-1, H-300, K-19, LK1), BD Biosciences (San Diego, CA, USA; clone 24/HSP60), Novus (Littleton, CO, USA; clone Mab11-13), Lifespan (Seattle, WA, USA; clone HSPD1), and Thermo Scientific (Rockford, IL, USA; clone 4B9/89). Isotype controls were from Cell Signalling, recombinant human Hsp60 from Loke Aps Diagnostics (Risskov, Denmark), recombinant mouse and rat Hsp60 from StressGen Biotechnologies (Victoria, BC, Canada), and recombinant hamster Hsp60 from IMMPACT Biotechnologies GmbH (Hamburg, Germany). For inhibition studies, the specific ERK1/2 inhibitor (PD98059) and the NF*κ*B inhibitor SN50 (Calbiochem, Darmstadt, Germany) were used.

### 2.4. Hsp60 Binding and Inhibition Studies

For Hsp60 binding studies, 0.5 × 10^6^ C57BL/6J, NOD, or NZO mouse-derived (pre-)adipocytes were either directly incubated with fluorescent-labelled Hsp60 (100 nM, Hsp60*) (45 min, 4°C) or preincubated with a tenfold molar excess of unlabelled Hsp60 from different species or ovalbumin (OVA, Sigma-Aldrich, Steinheim, Germany) as described [35]. For identification of Hsp60 binding epitopes, Hsp60* (100 nM) was preincubated with 0–25 *μ*g/mL of anti-Hsp60 antibodies (45 min, 4°C) before application to (pre-)adipocytes. After washing and fixation of the cells in 2% paraformaldehyde, Hsp60* binding was calculated from the geometric mean fluorescence after subtracting the autofluorescence as determined by analyses in a FACS Calibur flow cytometer (BD Biosciences).

### 2.5. Release of Inflammatory Mediators

C57BL/6J, NOD, and NZO mouse-derived (pre-)adipocytes were exposed to medium or 1–20 *μ*g/mL recombinant human Hsp60 (ENZO Life Sciences, Lörrach, Germany). After 24 h, the concentrations of the inflammatory mediators interleukin-6 (IL-6), mouse chemokine CXCL-1 (KC), and monocyte chemoattractant protein-1 (MCP-1) were determined in the culture supernatants by multiplex-beads-systems (Luminex Corp., Austin, TX, USA).

### 2.6. Analyses of Signaling Pathways

To identify Hsp60-activated signaling pathways, C57BL/6J, NOD, and NZO mouse-derived (pre-)adipocytes were seeded in 6 cm petri dishes (2 × 10^6^ cells/5 mL medium). Cells were treated with medium or Hsp60 (10 *μ*g/mL) (15 min, 37°C) and subsequently washed and treated with lysis-buffer (50 mM Tris-HCl, 150 mM NaCl, 1% NP-40, 0.25% Na-Desoxycholat) including protease and phosphatase inhibitors (Roche, Mannheim, Germany) (20 min, 4°C). After centrifugation (15 min, 10,000 ×g, 4°C) cell lysates were subjected to SDS-PAGE (10%) and appropriate antibodies for detection of activated signal proteins were applied for immunoblot analysis. Signals were visualized by the Lumi-Imager system (Roche Applied Science, Mannheim, Germany). To analyse the effect of specific signal protein inhibitors on the Hsp60-mediated secretion of inflammatory mediators, 1 × 10^5^ C57BL/6J, NOD, and NZO mouse-derived (pre-)adipocytes were seeded in 1 mL per well of a 48 well plate and incubated (1 h, 37°C) either with medium (control), ERK1/2 inhibitor PD98059 (100 *μ*g/mL), or NF*κ*B inhibitor SN50 (50 *μ*g/mL). Afterwards, cells remained unstimulated (medium) or were stimulated with Hsp60 (10 *μ*g/mL) for 24 h. Supernatants were collected to determine the concentrations of IL-6, KC, and MCP-1 by multiplex-beads-assays.

### 2.7. Statistical Analysis

Data were expressed as means ± SEM. Statistical analysis was performed using the Student's *t*-test. Differences were considered statistically significant with *P* < 0.05.

## 3. Results 

### 3.1. Hsp60 Binds Specific to C57BL/6J, NOD, and NZO Mouse-Derived Primary (Pre-)Adipocytes

**Figure 1:**
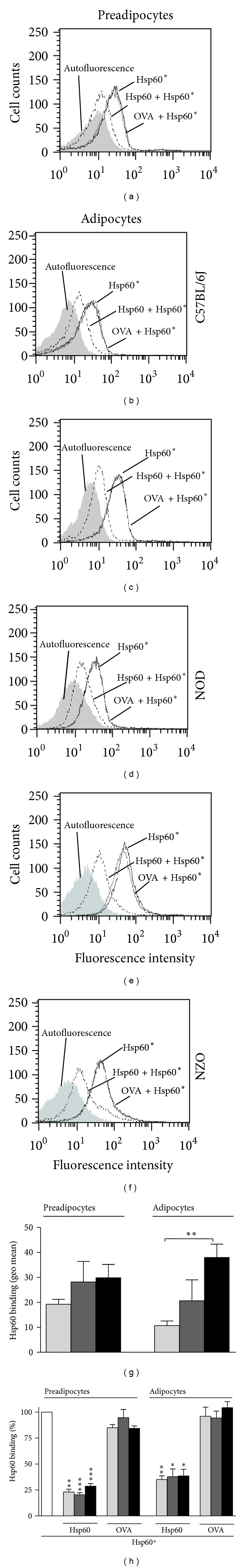
Hsp60 binding to C57BL/6J, NOD, and NZO mouse-derived (pre-)adipocytes. Preadipocytes (a, c, e) or adipocytes (b, d, f) of the three mouse strains were incubated with 100 nM DyLight649-labeled Hsp60 (Hsp60*) in the absence or presence of 1 *μ*M unlabeled Hsp60 or OVA. Binding of Hsp60* was quantified by determining the mean fluorescence intensities (geo mean) of the cell populations using FACS analysis. The data show representative results of Hsp60* binding and inhibition studies (a–f) and of Hsp60* binding to C57BL/6J (light grey bars), NOD (dark grey bars), and NZO mouse-derived cells (black bars) as means + SEM of three independent experiments (g). The specificity of Hsp60* binding was proven by analyses of the effects of unlabelled Hsp60 or OVA (h). The data represent residual Hsp60* binding to C57BL/6J (light grey bars), NOD (dark grey bars), and NZO mouse-derived cells (black bars) as percent of Hsp60* binding (set as 100%, white bar) in the absence of Hsp60 or OVA. The data show means + SEM of three independent experiments. *P < 0.05, **P < 0.01, ***P < 0.001 compared to other mouse strains (g) or compared to Hsp60* binding in the absence of Hsp60 or OVA (h).

To characterize the interaction between Hsp60 and primary (pre-)adipocytes from C57BL/6J, NOD, and NZO mice, we investigated the binding of fluorescent-labeled Hsp60 (Hsp60*, 100 nM) to preadipocytes and in vitro differentiated, mature adipocytes of the three mouse strains. FACS analyses demonstrated substantial binding of Hsp60 to adipocyte populations of all mouse strains (Figures 1(a)–1(f)). Comparison of the mean fluorescence signals revealed Hsp60 binding intensities of preadipocytes from C57BL/6J, NOD, and NZO mice within a range of geo means from 19 to 30 (Figure 1(g)). Among the adipocyte populations, NZO mouse-derived adipocytes revealed maximum Hsp60 binding (geo mean 38.0 ± 10.7).

The specificity of Hsp60-(pre-)adipocyte interaction was proven by inhibition of Hsp60* binding to cells preincubated with excess unlabelled Hsp60 (1000 nM) to 23.1 ± 2.7% (C57BL/6J), 20.4 ± 1.9% (NOD), and 28.7 ± 2.5% (NZO) (*P* < 0.05) (Figure 1(h)). Preincubation of the cells with OVA did not significantly affect Hsp60 binding. The extent of inhibition of Hsp60 binding to mature adipocytes was only marginally lower than that observed for preadipocytes.

### 3.2. Characterization of the Interaction of Different Hsp60 Species with C57BL/6J, NOD, and NZO Mouse-Derived (Pre-)Adipocytes

**Figure 2:**
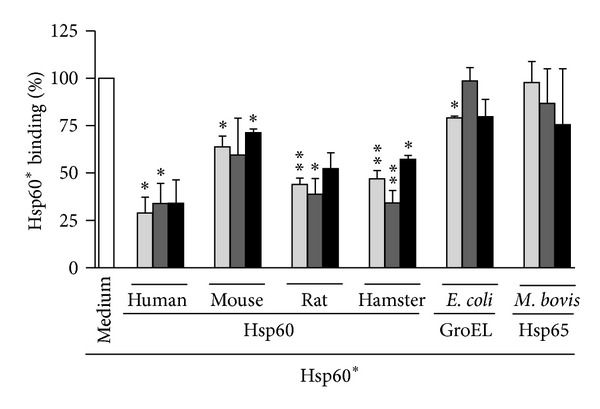
Characterization of the interaction of different Hsp60 species with preadipocytes from C57BL/6J, NOD, and NZO mice. C57BL/6J (light grey bars), NOD (dark grey bars), and NZO mouse-derived cells (black bars) were preincubated in the absence (Medium) or presence of 1 *μ*M unlabeled eukaryotic or prokaryotic Hsp60 species (human, mouse, rat, hamster, *E. coli,* and* M. bovis*), followed by addition of 100 nM human Hsp60-DyLight649 (Hsp60*). Hsp60* binding was quantified by FACS analysis. Data represent residual Hsp60* binding as percent of the binding of Hsp60* in the absence of Hsp60 or OVA (set as 100%, white bar). The results are shown as means + SEM of ≥ three independent experiments. *P < 0.05; **P < 0.01 compared to Hsp60* binding in the absence of Hsp60 and OVA.

Based on our previous observation that different eukaryotic Hsp60 species recognize the same receptor structure(s) on cells of the murine adipocyte line 3T3-L1 [35], we investigated the effect of eukaryotic (human, mouse, rat, and hamster) and prokaryotic Hsp60 species (*E. coli*, *M. bovis*) on the binding of human Hsp60 to primary C57BL/6J, NOD, and NZO mouse-derived (pre-)adipocytes. Strong inhibition of Hsp60* binding to preadipocytes was observed after preincubation of Hsp60* with the mammalian Hsp60 species (Figure 2). Preincubation with human Hsp60 induced maximum inhibitory effects by reducing Hsp60* binding to preadipocytes from the three mouse strains to 28.9 ± 14.5% (C57BL/6J), 33.9 ± 18.4% (NOD), and 34.1 ± 17.4% (NZO). Except for GroEL-mediated inhibition of Hsp60* binding to C57BL/6J mouse-derived preadipocytes, preincubation with prokaryotic Hsp60 species did not significantly affect Hsp60* binding to preadipocyte populations (Figure 2). For primary, in vitro differentiated adipocytes comparable results were obtained (data not shown).

### 3.3. Characterization of Hsp60 Binding Epitope(s) Involved in the Interaction with C57BL/6J, NOD, and NZO Mouse-Derived (Pre-)Adipocytes

Our further experiments focused on the identification of the Hsp60 epitope(s) potentially involved in the interaction of the stress protein with (pre-)adipocytes. In an initial approach, we investigated the effect of antibodies against distinct regions of the Hsp60 molecule, on the binding of Hsp60 to C57BL/6J, and NOD mouse-derived preadipocytes. FACS analyses revealed largely comparable patterns of antibody-mediated inhibition of Hsp60 binding to preadipocyte populations of both mouse strains (Table 1). Preincubation of the cells with antibodies against the N-terminal (aa1-50 (clone H1), aa1-200 (clone 24/HSP60)), and C-terminal (aa523-573 (clone K19)) regions of the Hsp60 molecule resulted in strongest reduction of Hsp60 binding to 25.5 ± 0.6%.

**Figure 3:**
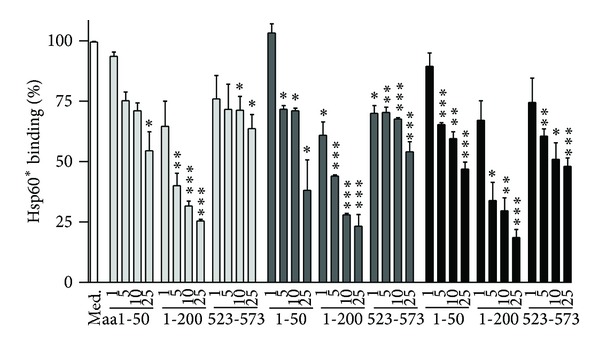
Identification of Hsp60 binding regions by Hsp60 epitope-specific antibodies. Increasing concentrations (0–25 *μ*g/mL) of antibodies directed against distinct aminoacid (aa) regions of the Hsp60 molecule were incubated with 100 nM Dylight649-labeled Hsp60 (Hsp60*) prior addition to C57BL/6J, NOD, or NZO mouse-derived preadipocytes. The resulting fluorescence signals of the cells were determined by FACS analysis. The data show residual Hsp60* binding to C57BL/6J (light grey bars), NOD (dark grey bars), and NZO mouse-derived cells (black bars) as percent of the binding of Hsp60* to cells cultivated in medium (Med.) in the absence of antibodies (set as 100%, white bar). The data represent means + SEM of three independent experiments. *P < 0.05; **P < 0.01; ***P < 0.001 compared to the binding of Hsp60* alone.

Subsequently, we preincubated fluorescent-labeled Hsp60 with increasing concentrations (0–25 *μ*g/mL) of the antibodies initially found to inhibit Hsp60 binding (clones H1, 24/HSP60, K-19 (Table 1)) prior addition to (pre-)adipocytes from C57BL/6J, NOD, or NZO mice (Figure 3). In C57BL/6J mouse-derived preadipocytes, Hsp60 binding was reduced dose-dependently to 51.2 ± 9.1% by the application of the antibody clone H-1 (aa1-50, 25 *μ*g/mL) and to 28.0 ± 2.6% by applying antibody clone 24/HSP60 (aa1-200, 25 *μ*g/mL). In contrast, clone K-19 (aa523-573) did not induce clear dose-dependent inhibition of Hsp60 binding. Comparable inhibitory effects were obtained for preadipocytes from NOD and NZO mice. Clone H-1 (aa1-50) reduced Hsp60 binding to 38.2 ± 12.5% (NOD) and to 47.0 ± 2.9% (NZO); clone 24/HSP60 (aa1-200) reduced Hsp60 binding to 23.3 ± 4.9% and to 18.6 ± 3.5% in NOD and NZO mouse-derived cells, respectively. As in C57BL/6J preadipocytes, no clear dose-dependent effects were observed for NOD and NZO mouse-derived preadipocytes preincubated with the antibody against the C-terminal region of Hsp60 (clone K-19, aa523-573). In binding studies with mature adipocytes, the antibodies yielded similar inhibition patterns (data not shown).

### 3.4. Hsp60-Induced Release of Inflammatory Mediators by C57BL/6J, NOD, and NZO Mouse-Derived Adipocyte Populations

**Figure 4:**
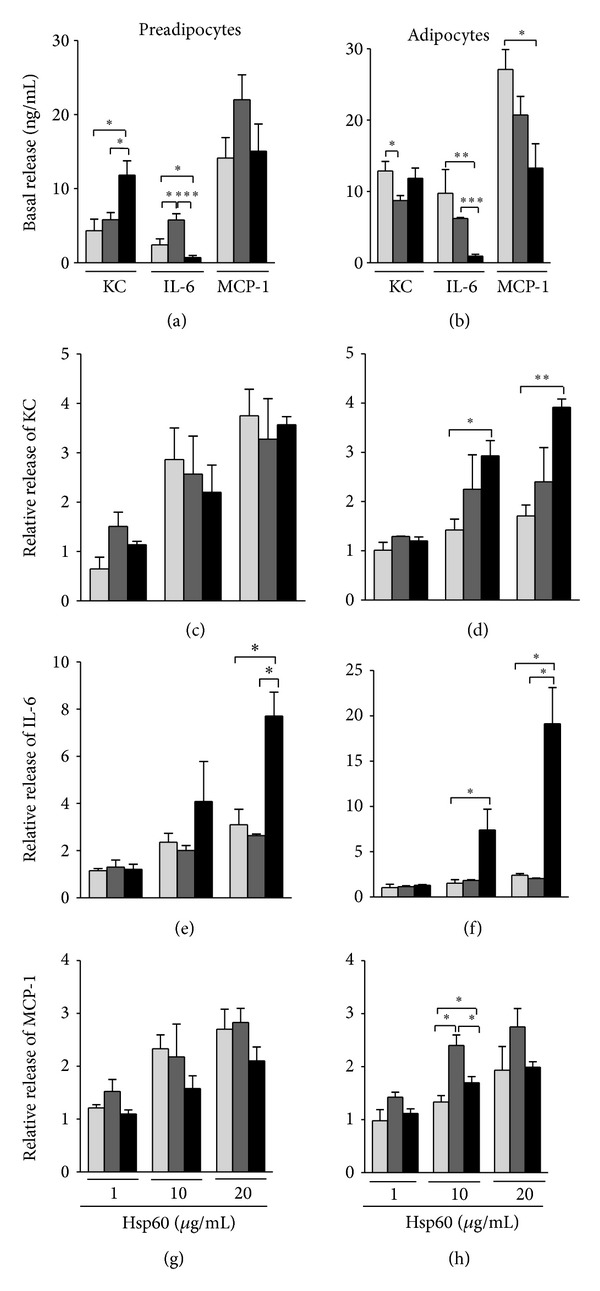
Hsp60-induced release of inflammatory mediators by (pre-)adipocytes from C57BL/6J, NOD, and NZO mice. (Pre-)adipocytes from C57BL/6J (light grey bars) NOD (dark grey bars), and NZO mice (black bars), were incubated in the absence (a, b) or presence (c–h) of increasing concentrations of Hsp60 (1–20 *μ*g/mL) and the accumulation of KC, IL-6, and MCP-1 in the culture supernatants was analyzed by multiplex-beads-assay after 24 h. The results show concentrations of accumulated cytokines (a, b) or of cytokine release relative to the cytokine levels detected in the supernatants of unstimulated cells (c–h). The data represent means + SEM of three independent experiments, each performed in triplicates. *P < 0.05, **P < 0.01, ***P < 0.001 compared to other mouse strains.

To compare adipocyte populations from different mouse models of diabetes for their ability to release inflammatory mediators, we investigated the accumulation of KC, IL-6, and MCP-1 in cultures of (pre-)adipocytes derived from C57BL/6J, NOD, and NZO mice (Figure 4). Unstimulated cells of the three mouse strains spontaneously accumulated substantial amounts of most of the mediators in their supernatants (Figures 4(a) and 4(b)). Independent of the mouse strain, preadipocyte and adipocyte populations released KC in a concentration range of 4.3–12.9 ng/mL and MCP-1 in a range of 13.3–27.1 ng/mL. The amounts of IL-6 released from C57BL/6J and NOD mouse-derived (pre-)adipocytes were in a range of 0.7–9.8 ng/mL, whereas (pre-)adipocytes from NZO mice released exceptionally low levels of the cytokine (0.7 ± 0.3 ng/mL and 0.9 ± 0.3 ng/mL IL-6, resp.) (*P* < 0.05).

Exposure of the (pre-)adipocyte populations to rising Hsp60 concentrations (1–20 *μ*g/mL) for 24 h caused a dose-dependent increase of the secretion of KC, IL-6, and MCP-1 in a cell type- and mouse strain-specific manner (Figures 4(c)–4(h)). However, whereas the cytokine release from NOD mouse-derived cell populations largely resembled the pattern of C57BL/6J mouse-derived cells, adipocyte populations from NZO mice showed an enhanced release of KC and IL-6. In particular, a strong, 3.9 ± 0.2-fold increase of KC release from NZO mouse-derived adipocytes was observed when compared to adipocytes from C57BL/6J (1.7 ± 0.2-fold increase, *P* < 0.05)) and from NOD mice (2.4 ± 0.7-fold increase) (Figure 4(d)). Moreover, the release of IL-6 induced by Hsp60 (20 *μ*g/mL) was strikingly increased from NZO mouse-derived preadipocytes (7.7 ± 1.0-fold) and adipocytes (19.1 ± 4.0-fold) when compared to the corresponding cell populations of C57BL/6J and NOD mice (*P* < 0.05) (Figures 4(e) and 4(f)).

### 3.5. Characterization of Hsp60-Activated Signaling Pathways in C57BL/6J, NOD, and NZO Mouse-Derived (Pre-)Adipocytes

**Figure 5:**
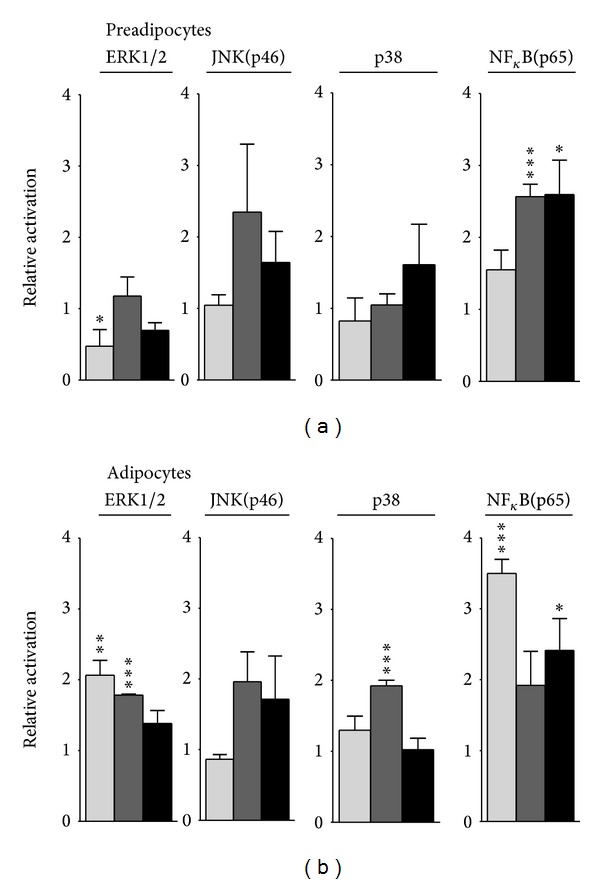
Hsp60 activates proinflammatory signaling in C57BL/6J, NOD, and NZO mouse-derived (pre-)adipocytes. Preadipocytes (a) and mature adipocytes (b) from C57BL/6J (light grey bars), NOD (dark grey bars), and NZO mice (black bars) were exposed to Hsp60 (10 *μ*g/mL) and after 15 min the activation (phosphorylation) of the MAP-kinases ERK1/2, p38, JNK(p46), and the transcription-factor NF*κ*B was analyzed by immunoblotting. The results show the activation levels of the signaling molecules relative to their activation in cells incubated in the absence of Hsp60. Data represent means + SEM from three independent experiments. *P < 0.05, **P < 0.01; ***P < 0.001 compared to medium control set as 1.

We further investigated the effect of Hsp60 on the stimulation of proinflammatory signaling pathways by analyzing the activation (phosphorylation) of members of the MAP kinase family (ERK1/2, JNK(p46), p38) and of the transcription factor NF*κ*B in (pre-)adipocytes of C57BL/6J, NOD, and NZO mice (Figure 5). Hsp60 treatment did not activate ERK1/2, JNK(p46), and p38 in preadipocytes of the three mouse strains (Figure 5(a)). On the other hand, NF*κ*B activation was increased to a similar degree in NOD and NZO mouse-derived preadipocytes (2.6 ± 0.2-fold and 2.6 ± 0.5-fold, resp.) (*P* < 0.05) but not in C57BL/6J mouse-derived cells. In contrast to the Hsp60 effects observed in preadipocytes, Hsp60 exposure of mature adipocytes increased the activation of ERK1/2 (2.1 ± 0.2-fold, *P* < 0.01) and NF*κ*B (3.5 ± 0.2-fold, *P* < 0.01) in C57BL/6J mouse-derived cells, whereas in cells from the diabetes predisposed mouse strains, Hsp60-induced activation of the investigated pathways was only moderate or low (<2-fold) (Figure 5(b)). These results show that Hsp60 preferentially controls ERK1/2- and NF*κ*B-dependent pathways in a differential manner depending on the maturation state of the adipocytes and on the donor mouse strain. We therefore focused our further analyses on the involvement of ERK1/2 and NF*κ*B in Hsp60-induced cytokine release by the use of specific inhibitors of the signaling molecules.

**Figure 6:**
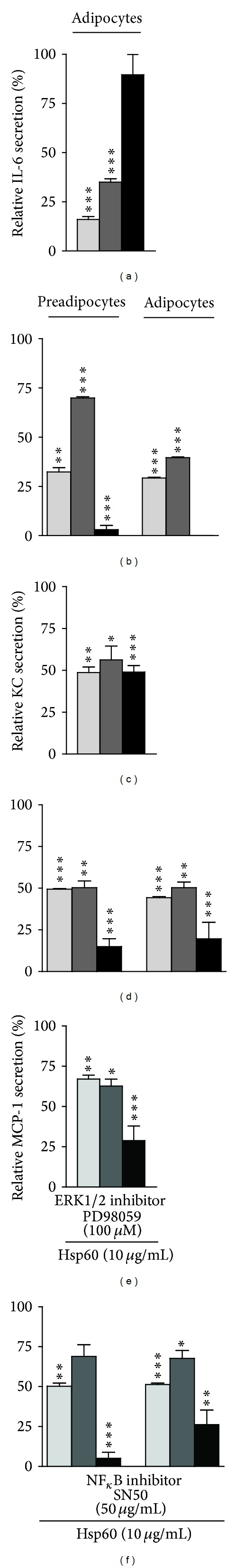
Characterization of signaling pathways involved in Hsp60-mediated release of inflammatory mediators from C57BL/6J, NOD, and NZO mouse-derived (pre-)adipocytes. Cells from C57BL/6J (light grey bars), NOD (dark grey bars), and NZO mice (black bars) were exposed to Hsp60 after preincubation (1 h) with specific inhibitors against the MAP-kinase ERK1/2 (PD98059) (a, c, e) and NF*κ*B (SN50) (b, d, f). After 24 h, IL-6 (a, b), KC (c, d), and MCP-1 (e, f) concentrations were determined in the culture supernatants by multiplex-beads-assay. The results show residual cytokine levels in percent of the cytokine concentrations released from cells incubated in the absence of Hsp60. Data show means + SEM from three to six experiments for each inhibitor. *P < 0.05, **P < 0.01, ***P < 0.001 compared to incubation with Hsp60 alone set as 100%.

Exposure of adipocytes of C57BL/6J and NOD mice to the ERK1/2-inhibitor PD98059 reduced the Hsp60-induced release of IL-6, KC, and MCP-1 to 16 to 67% (*P* < 0.05) of the levels detectable in cultures treated with Hsp60 alone (Figures 6(a), 6(c), and 6(e)). However, in NZO mouse-derived adipocytes, the Hsp60-induced cytokine pattern showed a differential response to ERK1/2 inhibition, whereas in the presence of PD98059, the formation of IL-6 remained largely unaffected (relative IL-6 secretion 89.7 ± 10.9%) (Figure 6(a)), the residual KC formation was in the range of the levels detected in C57BL/6J and NOD mouse derived cells (48.7 ± 3.8%, *P* < 0.001) (Figure 6(c)) and the release of MCP-1 was strongly reduced to 28.7 ± 9.1% (*P* < 0.001) (Figure 6(e)). Inhibition of NF*κ*B by SN50 resulted in the reduction of cytokine release in a similar range in cultures of (pre-)adipocytes from C57BL/6J and NOD mice (29–70%) (*P* < 0.01) (Figures 6(b), 6(d), and 6(f)). The strongest inhibition of Hsp60-induced IL-6, KC, and MCP-1 release was found in SN50-exposed (pre-)adipocytes from NZO mice. In NZO mouse-derived preadipocytes, the NF*κ*B inhibitor significantly reduced IL-6 release to 3.0 ± 2.1%, KC release to 15.0 ± 4.7%, and MCP-1 release to 5.1 ± 3.6% (*P* < 0.001) of the cytokine levels measured in the absence of the inhibitor. In adipocytes, NF*κ*B inhibition reduced IL-6 release to less than 0.1%, KC release to 19.7 ± 9.8%, and MCP-1 release to 26.3 ± 9.1% (*P* < 0.001).

## 4. Discussion

Previous observations suggest an essential contribution of obesity-associated inflammatory processes to the development of type 2 diabetes [10, 11] as well as type 1 diabetes [5]. Further studies, based on the finding of the strong immunomodulatory capacity of adipocytes, identified the stress protein Hsp60 as a potent inductor of proinflammatory mediators from murine and human adipocytes [21, 31]. These findings implicate that Hsp60-induced adipocyte mediators promote the development of obesity- and diabetes-associated inflammatory processes and further raise the question whether adipocytes from diabetes-prone subjects exhibit an aberrant reactivity to the stress protein.

To address this issue, we examined the effect of Hsp60 on adipocyte populations derived from the currently best characterized animal models of the two prevailing forms of human diabetes. Diabetes observed in the NOD mouse reflects major features of human type 1 diabetes [32, 33], whereas the metabolic abnormalities developing in the NZO mouse largely resemble the dysregulations of energy metabolism and glucose homeostasis associated with human type 2 diabetes [34]. As reference, adipocytes from the metabolically healthy mouse strain C57BL/6J were used. For our studies, we selected adipocyte populations isolated from the visceral fat depot as adipose tissue from this anatomical location had been identified as a major source of mediators responsible for diabetes-associated metabolic and immunologic disorders [36, 37]. As previous reports indicate that the functional properties of adipocytes depend on their maturation state [38, 39], we investigated Hsp60 effects on preadipocytes as well as terminally differentiated adipocytes.

Our approach focused on the decisive steps assumed to be involved in Hsp60-mediated inflammatory adipocyte activation: (1) the binding of Hsp60 to adipocytes regarded as the primary event in Hsp60-adipocyte interaction, (2) the activation of intracellular signaling pathways, and (3) the resulting release of inflammatory mediators which may act as signaling molecules in an autocrine manner and/or in the attraction and stimulation of immune cells.

Initial comparative FACS studies with fluorescent-labeled Hsp60 revealed that preadipocytes and mature adipocytes of all investigated mouse strains are able to bind Hsp60. Binding of the stress protein was highly specific as demonstrated by efficient inhibition after preincubation of the cells with unlabelled Hsp60. Interestingly, the observed characteristics of Hsp60-(pre-)adipocyte interaction showed striking similarities with Hsp60 binding by macrophages which was investigated in more detail in previous studies [40, 41]. These analyses demonstrated specific, receptor-mediated Hsp60 binding to the cell surface. Further studies revealed that the receptor structure for Hsp60 acts in a stereospecific manner, includes at least two functionally different components engaged in binding and signal transduction, differs from receptors for other heat shock proteins, and triggers endocytosis of its bound ligand. Considering the observed similarities in Hsp60 binding to (pre-)adipocytes and macrophages and in view of the close relationship between cells of the adipocyte and macrophage lineages [42], it may be concluded that Hsp60 binding to adipocytes is mediated by similar receptor-mediated mechanisms as the above mentioned mechanisms described for macrophages.

Interestingly, although efficient Hsp60 binding to adipocytes of all three mouse strains was observed, we found strongly enhanced binding of the stress protein to mature NZO mouse-derived adipocytes when compared to cells from C57BL/6J (and NOD) mice of the same maturation state. This finding may be of special importance in view of the fact that NZO mice are characterized by an increased body fat mass which most likely includes a large proportion of terminally differentiated adipocytes [43].

In more detailed studies, we attempted to identify Hsp60 epitopes involved in the interaction of the stress protein with (pre-)adipocytes. Competition experiments with various Hsp60 species and with inhibitory antibodies directed against defined Hsp60 epitopes revealed that eukaryotic Hsp60 molecules bind to (pre-)adipocytes via highly conserved aminoacid sequences within the N-terminal region.

Collectively, our studies on Hsp60-(pre-)adipocyte interaction demonstrate efficient Hsp60-binding to primary NOD mouse-derived adipocytes, thereby extending our previous observations of specific Hsp60 interaction with adipocytes from NZO mice and with cells of the murine adipocyte line 3T3-L1 [22]. Hence, our findings provide further evidence for the assumption that the ability to bind Hsp60 represents a general property of murine adipocytes irrespective of their origin and differentiation state. Nevertheless, further extensive biochemical studies will be necessary to gain further insight into the structural and functional properties of the Hsp60 receptor structure on (pre-)adipocytes.

Previous studies identified stress proteins as potent inducers of intracellular signaling pathways. We therefore investigated the effects of Hsp60 on the activation of the MAP kinases ERK1/2, JNK, and p38 and of the transcription factor NF*κ*B which are preferentially activated by stress signals but are also essential for the coordination of adipocyte differentiation [44–46]. Due to the marked interdependence of signaling and differentiation pathways, we hypothesized that the response of the adipocyte populations depends on the maturation state of the cells. In fact, Hsp60 exposure induced increased ERK1/2 activation in (mature) adipocytes from normal control C57BL/6J mice and enhanced activation of ERK1/2 and p38 in adipocytes from NOD mice. In contrast, Hsp60-exposed preadipocytes did not reveal consistent activation of MAP kinases. With regard to NF*κ*B, we found an elevated activation of the transcription factor in preadipocytes from diabetes-prone NOD and NZO mice but reduced activation in mature adipocytes of the two mouse strains, when compared to the reactivity of cell populations from normal control C57BL/6J mice. As NF*κ*B represents an important transcriptional regulator of inflammatory mediators [47], our findings implicate that preadipocytes of NOD and NZO mice exhibit an increased Hsp60 responsiveness that might contribute to diabetes-promoting proinflammatory processes in these animals which are genetically predisposed to develop diabetes.

Collectively, our findings implicate that the Hsp60-induced activation of intracellular signaling pathways in adipocytes not only depends on the differentiation state of the cells but also on the donor mouse strain. Our observations particularly suggest that aberrant intracellular signaling and disturbed transcriptional regulation in adipocytes enhance diabetes-promoting inflammatory reactivity in animals with a genetic predisposition to develop diabetes. In fact, previous studies demonstrated aberrant activation of MAP kinases, including p38, [48, 49] and a dysregulation of transcription factors, including NF*κ*B [50–52] in diabetes-prone mouse strains. Although these findings are mainly derived from macrophages, they obviously reflect intrinsic disturbances of signaling and transcriptional processes in diabetes prone NOD and NZO mice that may contribute to the aberrant activation of MAP kinases and NF*κ*B observed in Hsp60-exposed (pre-)adipocytes of these animals. Further studies on the elucidation of the intracellular signaling cascade triggered by stress signals (will) have to consider the complex situation that most components of the stress response pathways are also critical for the control of adipocyte differentiation [45, 50–52].

The release of proinflammatory adipocyte mediators induced by Hsp60 and the intracellular signaling pathways engaged in this process were further analyzed by focusing on the formation of KC, IL-6, and MCP-1 which had been identified as prominent factors released by adipocytes and are supposed to contribute to obesity-associated inflammatory processes [53–55]. Unstimulated preadipocytes and adipocytes of C57BL/6J, NOD, and NZO mice released substantial amounts of these mediators, with the exception of NZO mouse-derived adipocyte populations which released markedly low amounts of IL-6. Hsp60, applied at concentrations that largely correspond to the levels found in individuals suffering from cardiovascular and arthritic disorders [56, 57], dose-dependently increased the release of KC, IL-6, and MCP-1 by preadipocytes and adipocytes from the three mouse strains. These results extend our initial observations on the pronounced Hsp60 responsiveness of adipocytes of a murine cell line and of NZO mice [21, 22] and further support the view that the capacity to release proinflammatory mediators in response to Hsp60 reflects a common feature of adipocytes. However, with regard to the release of IL-6, inducibility by Hsp60 was strikingly higher in NZO mouse-derived adipocyte populations than in cells from C57BL/6J and NOD mice.

The potential impact of the donor mouse strain on MAP kinase-dependent signaling and transcriptional regulation in the Hsp60-induced formation of inflammatory adipocyte mediators was further elucidated by selective inhibition of the signaling molecules. Blocking of signaling pathways by specific inhibitors [58, 59] confirmed the involvement of ERK1/2 and NF*κ*B in the Hsp60-induced release of adipocyte mediators. Unexpectedly, the experiments revealed strong differences between the reactivities of adipocyte populations from the two diabetes-predisposed mouse strains. After ERK1/2 or NF*κ*B inhibition, the reactivity pattern of NOD mouse-derived cells largely resembled the pattern of cells from normal control C57BL/6J mice. However, in adipocyte populations of NZO mice, ERK1/2 dependency was low for IL-6 production, intermediate for KC production, and high for MCP-1 production when compared to C57BL/6J and NOD mouse-derived cells. Moreover, NF*κ*B suppression caused strongest inhibition of 75–98% in cultures of NZO mouse-derived cells pointing to a dominant role of NF*κ*B in the formation of proinflammatory mediators from Hsp60-exposed adipocytes in this mouse strain. This finding appears to be in discrepancy with our above-mentioned results demonstrating that NOD and NZO mouse-derived adipocytes show largely comparable activation levels of MAP kinases and NF*κ*B after Hsp60 exposure. These contrasting observations obviously reflect strain-specific differences in the preferential usage of intracellular pathways of signaling and transcriptional regulation (other than NF*κ*B-dependent) to induce the formation of inflammatory mediators in response to Hsp60. This assumption is supported by the finding of aberrant activation of intracellular signaling pathways in diabetes-prone mouse strains [49, 51, 52]. Furthermore, the promoter regions of many genes encoding inflammatory mediators (e.g, IL-6, MCP-1) also have binding sites for other transcription factors including activator protein 1 and members of the CCAAT-enhancer binding protein family [60–62]. It might be speculated that these factors could be used alternatively to NF*κ*B in the induction of proinflammatory adipocyte mediators in different mouse strains, depending, for example, on their genetic predisposition to develop a specific form of diabetes.

Taken together, our observations in C57BL/6J, NOD, and NZO mouse-derived adipocyte populations indicate that the basic structural requirements, such as receptor structure(s) and Hsp60 binding epitopes, involved in the initial recognition of the stress protein, share a high degree of similarity, and irrespective of the donor and of the differentiation state of the cells. However, clear differences between the mouse strains were found regarding the efficiency of Hsp60 binding, the patterns of intracellular signaling pathways activated by Hsp60, and the resulting release of inflammatory mediators. These findings warrant further extensive studies for the detailed elucidation of mouse strain dependency and the roles of intracellular signaling pathways in Hsp60-induced adipocyte stimulation.

NOD mice typically exhibit a lean constitution and, accordingly, a low number of (mature) adipocytes. Moreover, the Hsp60 responsiveness of NOD mouse-derived adipocytes largely corresponds to the reactivity of adipocytes of normal healthy C57BL/6J mice. Based on these considerations, it may be concluded that in NOD mice, Hsp60-induced proinflammatory adipocyte activities do not play a decisive role in the progression of (insulin deficient) diabetes. In NZO mice, however, improved Hsp60 binding and dysregulation of IL-6 formation reflect an increased proinflammatory reactivity, preferably of mature adipocytes. In addition, the pathological increase of the adipose tissue mass by adipocyte-hyperplasia and proliferation inevitably leads to an elevated number of (mature) adipocytes in these mice. As a result, the enhanced release of chemotactic and stimulatory adipocyte mediators may contribute not only to the acceleration of immune cell infiltration into the adipose tissue but also to an elevation of systemic inflammatory mediators which may aggravate peripheral insulin resistance, thereby promoting the progression of metabolic dysregulation and diabetes. Moreover, during the development of obesity, the expanding adipose tissue itself may create an increasingly stressful environment in which infiltrating immune cells (e.g, macrophages) and adipocytes themselves may become relevant sources of Hsp60 and other stress signals.

## Figures and Tables

**Table 1:** Inhibition of Hsp60 binding to C57BL/6J and NOD mouse-derived preadipocytes by antibodies directed against defined Hsp60 regions.

Anti-human Hsp60 antibody		Hsp60* binding (%)	
Clone	Recognized Hsp60 region (aa)	C57BL/6J preadipocytes	NOD preadipocytes
H1	1–50	54.5 ± 7.8*	46.2 ± 11.9*
24/HSP60	1–200	25.5 ± 0.6***	28.0 ± 6.1***
H-300	274–573	62.7 ± 5.2*	65.5 ± 1.8*
4B9/89	335–366	70.5 ± 7.5	78.8 ± 7.9
HSPD1	383–419	70.5 ± 1.7***	74.4 ± 1.7*
LK-1	383–447	63.8 ± 1.8*	53.6 ± 12.4
K-19	523–573	61.9 ± 4.0***	42.1 ± 7.1*

The binding of (fluorescent-labeled) Hsp60* (100 nM) in the absence of antibodies was set as 100%. The data represent residual Hsp60 binding in the presence of antibodies as mean ± SEM of three independent experiments. The application of adequate isotype controls to rule out unspecific inhibitory effects, showed no significant inhibition, whereas inhibition > 50% is regarded as biologically relevant, statistically significant inhibition of Hsp60* binding is indicated as *P < 0.05; ***P < 0.001.

## Abbreviations

CXCL-1/KC: Chemokine (C-X-C motif) ligand 1
ERK1/2: Extracellular signal-regulated kinase 1/2
Hsp(60): Heat shock protein (60)
JNK: c-Jun N-terminal kinase
MCP-1: Macrophage chemoattractant protein 1
MAPK: Mitogen-activated protein kinase
NF*κ*B: Nuclear transcription factor *κ*B
NZO: New Zealand obese
OVA: Ovalbumin
PBMC: Peripheral blood mononuclear cells
PBS: Phosphate buffered saline.
